# Different Cortex Activation and Functional Connectivity in Executive Function Between Young and Elder People During Stroop Test: An fNIRS Study

**DOI:** 10.3389/fnagi.2022.864662

**Published:** 2022-08-03

**Authors:** Wenhao Huang, Xin Li, Hui Xie, Tong Qiao, Yadan Zheng, Liujie Su, Zhi-Ming Tang, Zulin Dou

**Affiliations:** ^1^Department of Rehabilitation Medicine, The Third Affiliated Hospital of Sun Yat- sen University, Guangzhou, China; ^2^Key Laboratory for Biomechanics and Mechanobiology of Ministry of Education, School of Biological Science and Medical Engineering, Beihang University, Beijing, China; ^3^Affiliated Hospital of Inner Mongolia Medical University, Hohhot, China

**Keywords:** fNIRS (functional near infrared spectroscopy), cortex activation, functional connectivity, executive function, different age

## Abstract

**Objective:**

The objective of this study was to examine the activation and functional connectivity of the prefrontal and temporal lobe in young and elder people during the Stroop test using functional near-infrared spectroscopy (fNIRS).

**Methods:**

A total of 33 healthy volunteers (20 young people, mean age: 23.7 ± 3.9 years; 13 elder people, mean age: 63.9 ± 4.0 years) participated in the study. All subjects were asked to finish the Stroop Color Word Test. The oxygenated hemoglobin concentration (Delta [HbO_2_]) signals and the deoxygenated hemoglobin (Delta [HbR]) signals were recorded from temporopolar area (TA), pars triangularis Broca's area (Broca), dorsolateral prefrontal cortex (DLPFC), and frontopolar area (FA) by fNIRS. The coherence between the left and right frontotemporal lobe delta [HbO_2_] oscillations in four frequency intervals (I, 0.6–2 Hz; II, 0.145–0.6 Hz; III, 0.052–0.145 Hz; and IV, 0.021–0.052 Hz) was analyzed using wavelet coherence analysis and wavelet phase coherent.

**Results:**

In the Stroop test, the young group was significantly better than the elder group at the responses time, whether at congruent tasks or at incongruent tasks (congruent: *F* = 250.295, *p* < 0.001; incongruent: *p* < 0.001). The accuracy of the two groups differed significantly when performing incongruent tasks but not when performing congruent tasks (incongruent: *F* = 9.498, *p* = 0.001; congruent: *p* = 0.254). Besides, only elders show significant activation in DLPFC, Broca, FA, and TA (*p* < 0.05) during the Stroop test, but young people did not show significant differences. In the functional connectivity of task states, younger people had stronger connections between different brain regions in both the left and right brain compared with the elderly (*p* < 0.05). In particular, the left and right DLPFC showed stronger connection strength in most of the brain areas. The result suggested that younger people had stronger functional connectivity of brain areas than older people when completing the task.

**Conclusion:**

According to these results, although the cortical activation in the elder people was higher than the young people, the young showed stronger connectivity in most of the brain areas than the elders. Both sides of DLPFC and right Broca area were the most significant cortical activation in Stroop test. It was suggested that the decrease in functional connectivity in the elder people resulted in the atrophy of white matter, to which we should pay more attention.

## Introduction

As our society ages, deteriorating cognitive function and linguistic abilities in the elderly are associated with increased mortality, hospitalization rates, and a decline in quality of life (Kim and Oh, 2013; Shankar et al., 2014). In recent years, there has been a rise in interest in the executive function, which is an essential component of cognitive function (Dong et al., 2010). Elder executive dysfunction is significant not just for healthcare professionals and social assistance agencies, but also for public health experts, community groups, and other important fields. According to previous studies, execution problems might affect daily living by interfering with the planning and timing of physical activities (O'Bryant et al., 2011; Ezzati et al., 2019). The adversity of executive impairment in the elderly may result in depression that modifies mental health-related quality-of-life indicators. Therefore, for a better understanding of executive dysfunction in the elderly, early diagnosis is required.

The Stroop Color Word Interference Test is an extremely significant cognitive neuroscience test (Zalonis et al., 2009; Chen and Li, 2012). It mainly uses the principle of the Stroop effect-psychology referring to the interference of the dominant response of the brain to the non-dominant response and is mainly used to evaluate executive function and language problem (Alvarez and Emory, 2006). In the test, the participants were asked to identify the words printed in different colors (such as “red” written in yellow), requiring the brain to simultaneously process color and text. The behavioral and neuroimaging experiments concluded that semantic rivalry caused interference in the color word Stroop task (Stroop, 1935).

It was discovered previously that healthy older persons are less effective at suppressing reaction activity primed by the fully processed meaning of the irrelevant word dimension (Stuss et al., 2001). The fMRI data revealed that age-related deterioration of executive function could develop as early as the age of 50 years and worsen by the age 60 years (Milham et al., 2002; Kaufmann et al., 2008; Mathis et al., 2009; Mohtasib et al., 2012). Compared to younger adults, older adults demonstrated stronger interference effects, and these interference effects increased as memory load rose (Bondi et al., 2002). Age has been linked to diminished cognitive performance. In fact, the Stroop test is one of the evaluations used to diagnose cognitive decline in the elderly.

Similar to fMRI, functional near-infrared spectroscopy (fNIRS) monitors changes in the kinds of hemoglobin in the brain using the differential in light absorption between oxygenated hemoglobin (oxy-Hb) and deoxygenated hemoglobin (deoxy-Hb) in the near-infrared (700–900 nm) (Chen et al., 2020; Huang et al., 2020). The simple and practical fNIRS can be used to conduct experiments in an open and quiet environment, thereby enhancing the effectiveness of the experiment in the experimental context. In addition, evidence from earlier fNIRS and fMRI papers corroborated the positive connection between cerebral oxygenation and Stroop performance in healthy older males (Ehlis et al., 2005; Bu et al., 2018a; Li et al., 2020). Initial neuroimaging investigations of inhibitory control revealed that older people had increased activation in various frontal regions, particularly the inferior frontal gyrus, indicating task-related recruitment (Zhang et al., 2017). However, a drawback of earlier publications was that the functional connection between young and old people was generally overlooked and the association between brain activation and functional connectivity was not well investigated.

To gain a better understanding of the impacts of aging, we evaluated the effects of brain activation and functional connectivity of young people and old Stroop test-takers. The frontal and temporal lobes were examined by fNIRS during the Stroop test, and there was increased interest in the brain connections of inhibitory control activities.

## Materials and Methods

### Subjects

A total of 33 healthy subjects, including 14 older adults (8 females), ages 60–72 years, and 20 young adults (12 females), ages between 18 and 35 years, were recruited for this study. All subjects met the following criteria, namely, (1) no personal or family history of neuropsychiatric disorders and no medication; (2) no history of alcohol abuse, no staying up late, or other conditions affecting mental and cognitive health; (3) normal vision and no color blindness; (4) all right-handed and no upper-limb problems; and (5) no obvious cognitive problems and live independently. The research was conducted at the Third Hospital of Sun Yat-sen University Hospital, China. All subjects agreed to participate and signed an informed consent form. The experimental methods were approved by the Ethics Committee of the Third Hospital of Sun Yat-sen University Hospital and conformed to the 1975 Declaration of Helsinki (revised in 2008). The registration number was ChiCTR2100053445, which was verified by the Chinese Clinical Trial Registry. Table 1 summarizes the characteristics of the subjects.

**Table 1:** The *p-*values of the comparison between task values and rest values for elder and young people and the *p-*values of task period between elder and young people (*p < 0.05).

**The brain areas**		**Elder-Task vs. Rest**	**Young-Task vs. Rest**	**Task-Elder vs. Young**
MTG	R-MTG	0.129	0.505	0.077
	L-MTG	0.285	0.715	0.114
STG	R-STG	0.236	0.505	0.537
	L-STG	0.124	0.98	0.076
TA	R-TA	0.846	0.657	0.92
	L-TA	0.068*	0.591	0.122
Broca	R-Broca	0.04*	0.461	0.151
	L-Broca	0.483	0.928	0.235
DPLFC	R-DPLC	0.032	0.82	0.715
	L-DPLC	0.026*	0.941	0.22
FA	R-FA	0.28	0.792	0.212
	L-FA	0.02*	0.771	0.122

### Equipment

**Figure 1:**
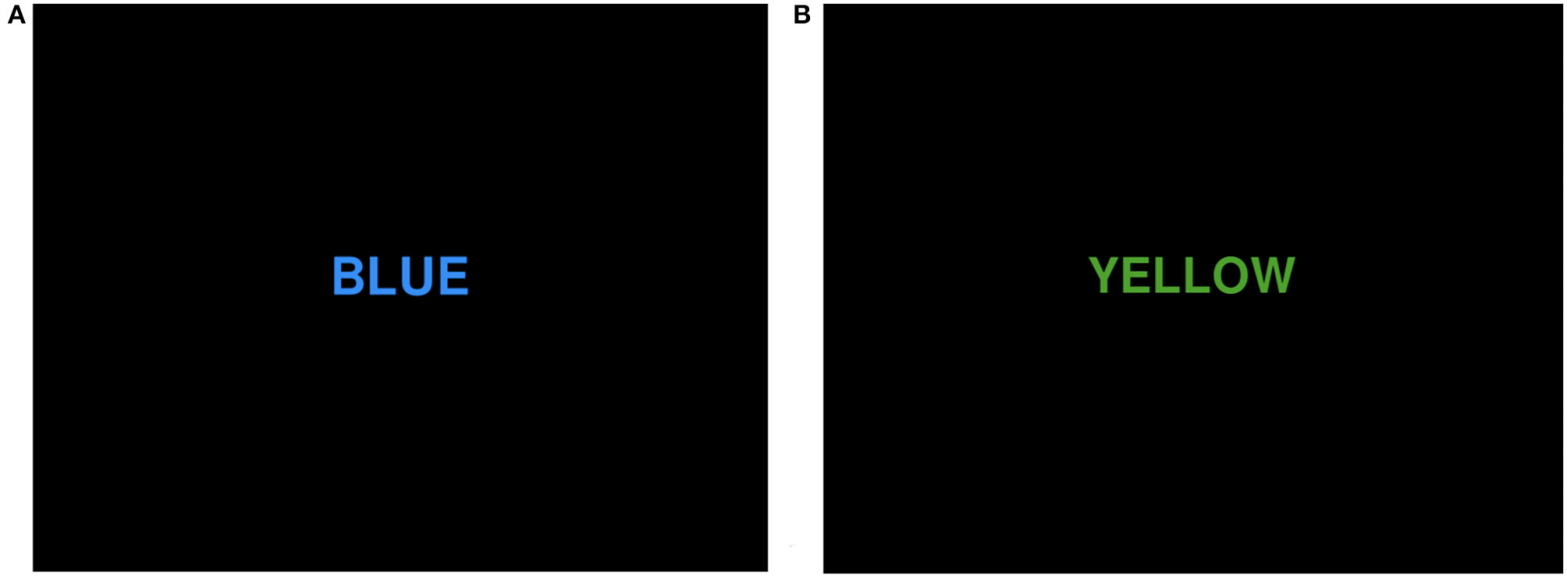
Two different types of Stroop tasks: **(A)** congruent task; **(B)** incongruent task.

Participants were instructed to sit in a 50-cm-high reclining chair in front of a table and computer of the proper height. The Stroop test consisted of two sections, i.e., one for congruent tasks and the other for incongruent tasks. In the congruent task, the color of the displayed words had the same significance as the words themselves. For instance, when the word “BLUE” showed on the screen, the word's color was also blue (Figure 1A). In an incongruent task, the meaning and color of the displayed words do not correspond. If the word “YELLOW” appears on the screen, for instance, the word's color is green (Figure 1B). The colors that emerge at random on the display are yellow, red, green, and blue. Whenever a new word appeared on the screen, participants were instructed to press the appropriate button on the QWERTY keyboard as rapidly and accurately as possible (Y = yellow, R = red, G = green, B = blue). When the participant clicked on the correct or incorrect button, a white cross pattern would be presented for 0.5 s as a break. If the subject did not push the button within 2 s, the computer would detect it as an error and the subject would receive a 0.5 s break. Each Stoop test consisted of 40 random word identifications that required approximately 90 s to complete.

### Experimental Procedure and Measurements

**Figure 2:**
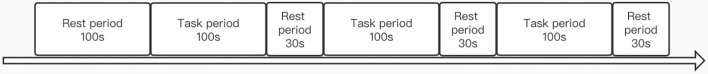
Task flow diagram of fNIRS.

The experiments were conducted using a computer and fNIRS. Experiments were conducted in a quiet area to reduce unwanted stimuli that could influence the testing of participants. Each participant was instructed to sit comfortably in a chair and relax his or her body. Before the formal beginning of the experiment, the subject would listen to the experimenter's explanation and make a preliminary attempt. When the subject was confident that he understood the entire task and procedure, he would take a brief break before beginning the formal experiment. Based on the findings of the previous experiment, the Nirscan software (supplied by the device manufacturer) was used to label the recorded targets during the trial. The Nirscan automatically captured the pertinent data for later analysis and processing. The entire experiment was carried out in two phases (Figure 2). The first portion consisted of a 100-s rest period during which the participant gazes at the white cross in front of him. The second section was a 390-s task phase consisting of three cycles of a 100-s Stroop test and a 30-s rest period. In each Stroop test, the computer automatically records the subject's correct and erroneous responses to each word, as well as the amount of time required to make a decision. After the tests were completed, the system generated the response time and accuracy of the Stroop test automatically.

### fNIRS Data Acquisition

**Figure 3:**
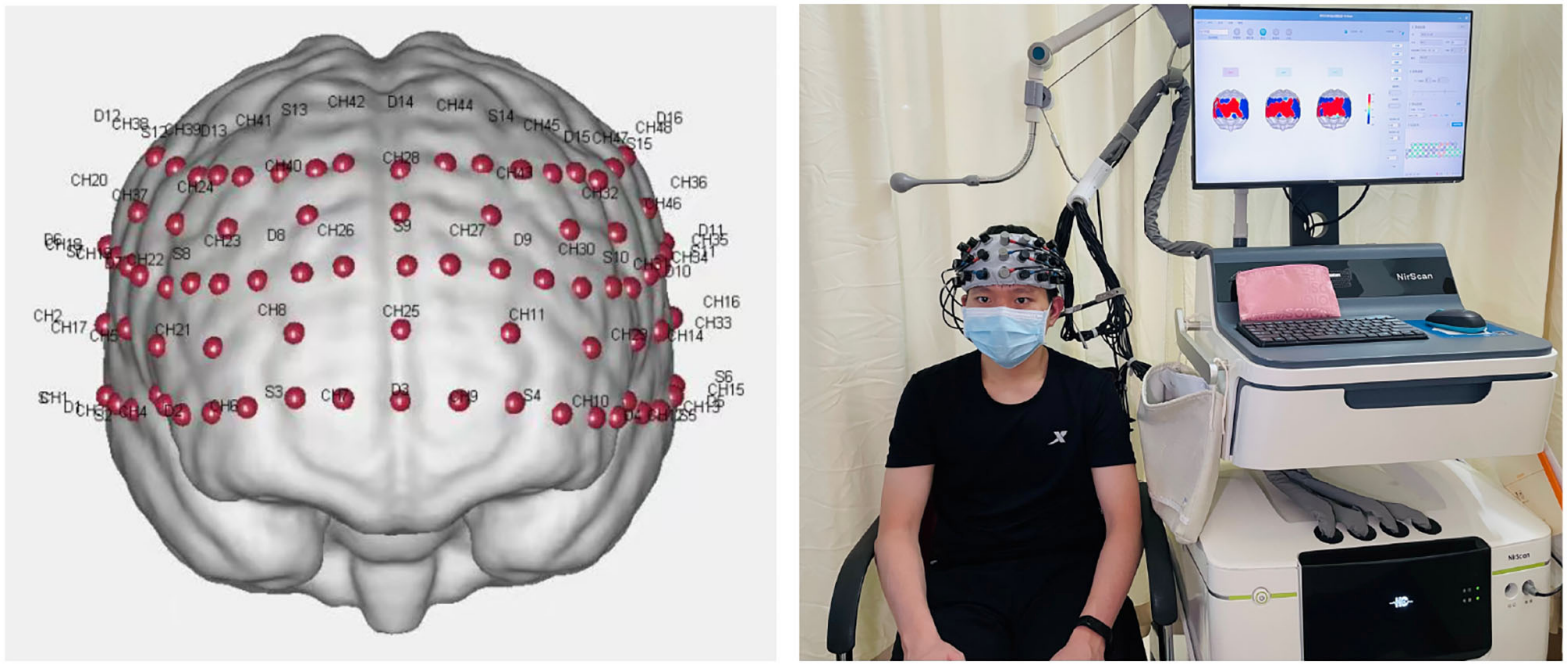
Distribution of channels in the frontotemporal lobe of the brain.

In the experiment, variations in HbO and HbR signals were recorded using a multichannel NIR system (Nirsmart, Danyang Huichuang Medical Equipment Co., Ltd, China). The program was built using 15 sources and 16 detectors, which comprise 48 effective channels in the experimental design (the device contains 24 sources and 24 detectors, which can surpass 80 effective channels) with a channel spacing of 3 cm. The reference to the international 10–20 system for positioning included the frontal, parietal, and temporal lobe regions, including the dorsolateral prefrontal cortex (DLPFC), middle temporal gyrus (MTG), superior temporal gyrus (STG), temporopolar area (TA), pars triangularis Broca's area (BROCA), and frontopolar area (FA) (Figure 3). All channels of the gadget sample at a rate of 11 Hz. The light source probe is utilized at wavelengths of 730 and 850 nm, and the detector is a very sensitive avalanche diode APD. To repair the emitter and collector, a head cap is used. Prior to the experiment, a pre-test was conducted to check that all channels were appropriately utilized.

### Data Preprocessing and Analysis

To analyze the data of fNIRS, moving-average filter and sixth-order Butterworth filter were used to eliminate abrupt singular points, low-frequency variations (<0.052 Hz), high-frequency noises (>2 Hz), and the baseline wanderings (Xu L. et al., 2017; Bu et al., 2018a). The calculation of a moving average was a signal processing technique that used an averaging method to increase the signal-to-noise ratio.

#### Wavelet Transform (WT)

WT is a transformation method of time series from the time domain to the frequency domain to obtain the main component of the time series in the frequency domain (Li et al., 2014). Tunable filter band lengths were used to provide the appropriate time and frequency resolution, which projected the time series onto the time–frequency plane, thereby obtaining the time–frequency amplitude 3D map. Morlet wavelet has its best time–frequency compactness and thus was used for continuous WT in this study. WT distinguished four frequency intervals, as follows: I: 0.6–2 Hz; II: 0.145–0.6 Hz; III: 0.052–0.145 Hz; and IV: 0.021–0.052 Hz. The physiological meanings of cardiac, respiratory, myogenic, and neurogenic activities processes are represented by the four frequency intervals (Landsverk et al., 2007; Tan et al., 2015; Kim et al., 2020). The results of WT were averaged over the time domain to obtain the wavelet analysis (WA) of each Delta [HbO_2_] and [HHb] signal at each time and frequency. WA is characterized by the intensity or activation of the cerebral cortex.

#### Wavelet-Based Coherence Analysis

This method was described in our previous studies (Bu et al., 2017, 2018a,b; Xu G. et al., 2017). Wavelet phase coherence (WPCO) was used to determine phase coherence by calculating the phase difference of signal after the WT. The result explained the change characteristics of the connection between different brain regions in terms of phase congruency. In this study, for each subject, WPCO valued among all possible pairs of 48 channels were calculated for the specific posture state and frequency intervals.

### Statistical Analysis

Using the G^*^Power statistical tool version 3.1, it was determined that a sample size was required to achieve a statistical power of 80% with statistical significance at *p* < 0.05 (two-tailed) and effect size of 0.4 based on preliminary experimental results (Milham et al., 2002; Langenecker et al., 2004; Plenger et al., 2016; Yennu et al., 2016). Statistical analysis of the data was performed using IBM SPSS (v. 26.0). Levene test was used to ensure that the WPCO values satisfied the assumption for parameter analysis. Two-way ANOVA (congruence × group) repeated measurements were conducted to compare the differences in mean Delta oxy-Hb and Delta deoxy-Hb changes and WPCO values in different states (rest and task). *Post-hoc* test was performed using Bonferroni comparison test. A difference of *p* < 0.0125 was considered statistically significant.

## Results

### The Data of Stroop Test

**Figure 4:**
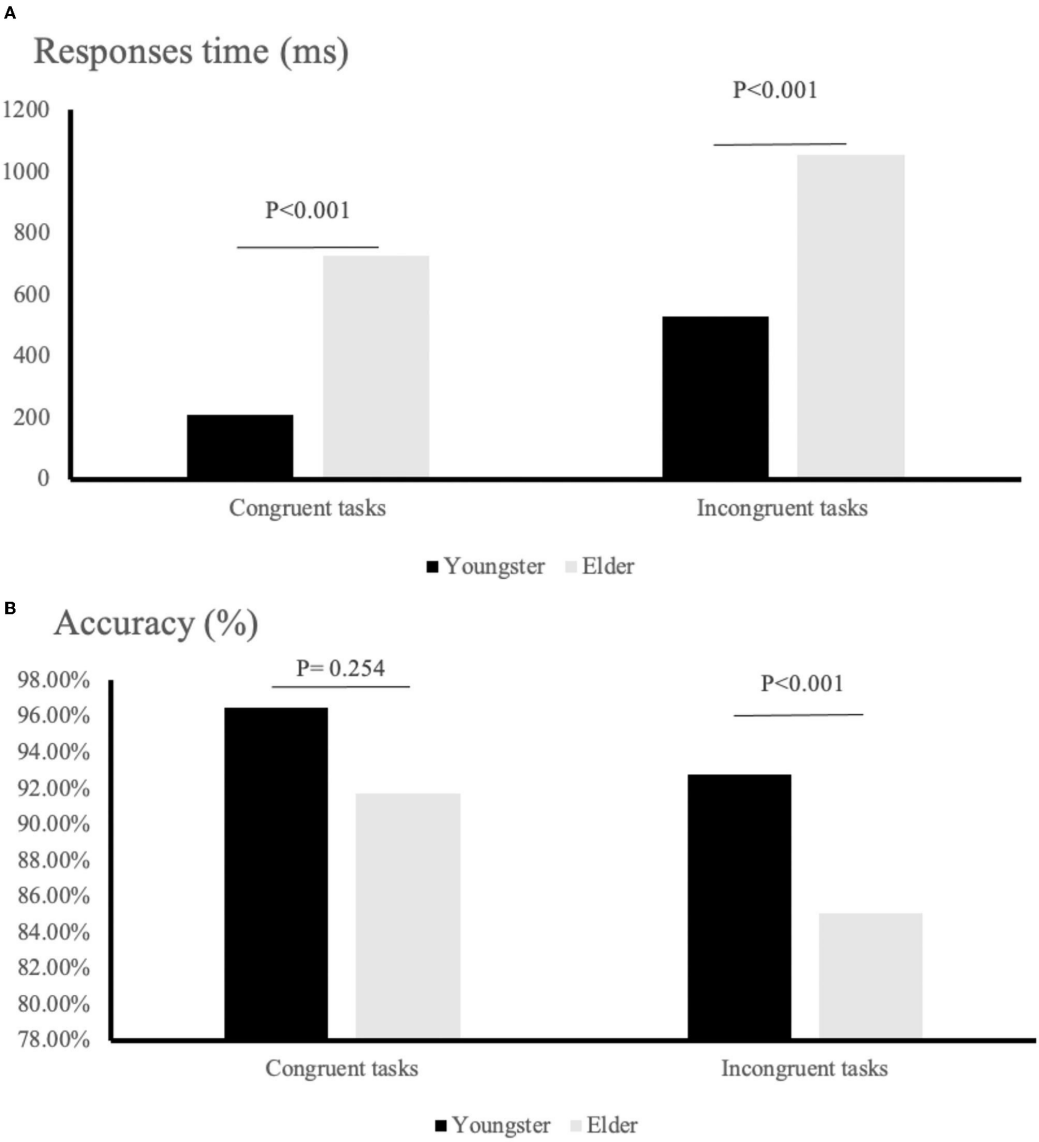
Behavior results for task performance: **(A)** response times; **(B)** rate of correct answer.

Figure 4 indicates the correct number and responses time when congruent and incongruent tasks were completed in both young and old participants. For youngsters, the correct rate for congruent activities was 96.49%, whereas the correct rate for incongruent tasks was 94.28%. In addition, the average response time for accurate answers to congruent and incongruent tests was 211 and 725 ms, respectively. For the elderly, the correct rate for congruent activities was 91.73%, whereas the correct rate for incongruent tasks was 85.07%. The average response time for accurate responses to congruent and incongruent tests was 529 and 1,053 ms, respectively.

The 2 ×2 repeated-measures ANOVA showed that the young group was significantly faster than the elder group in their responses, both in congruent and incongruent trials (congruent tasks: *F* = 250.295, *p* < 0.001; incongruent tasks: *p* < 0.001). In contrast, the accuracy of the two groups differed significantly when performing incongruent tasks but not when performing congruent tasks (incongruent tasks: *F* = 9.498, *p* = 0.001; congruent tasks: *p* = 0.254).

### The Data of fNIRS

#### Comparison of WA Between Task Period and Rest Period

**Figure 5:**
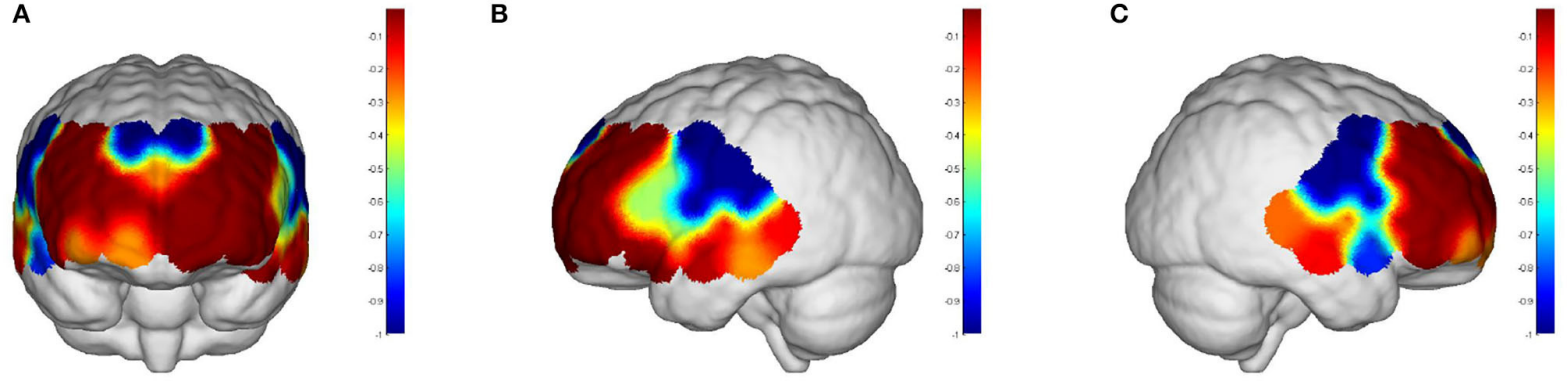
The *p-*values of the comparison between task values and rest values for older adults: **(A)** front view; **(B)** left view; **(C)** right view. But both the prefrontal and DLPFC showed a strong activation and were statistically significant (*p* < 0.05).

**Figure 6:**
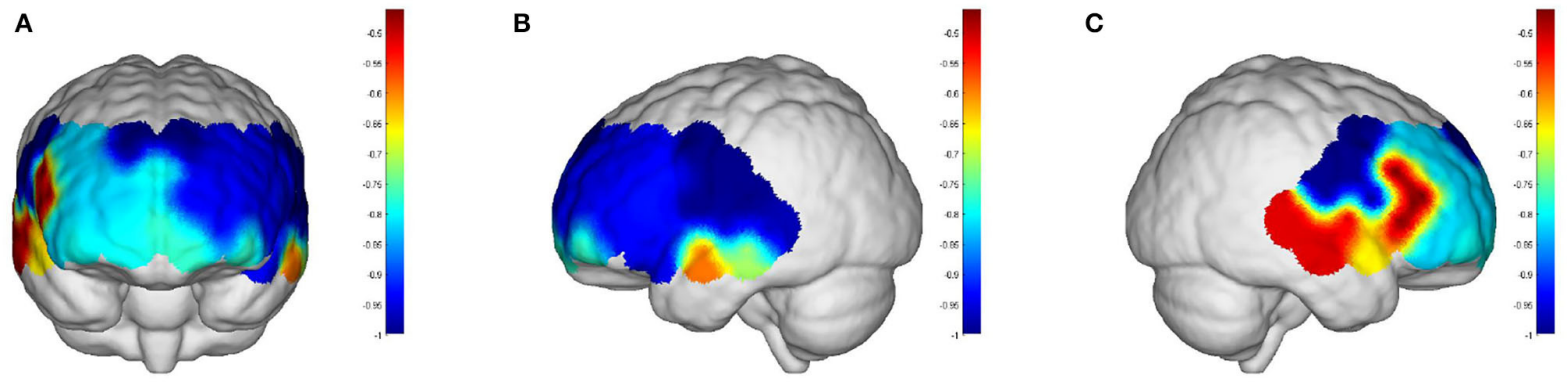
The *p-*values of the comparison between task values and rest values for young people: **(A)** front view; **(B)** left view; **(C)** right view. The red color represents stronger activation. The red areas are shown in DPLFC, which were *p* > 0.05.

**Figure 7:**
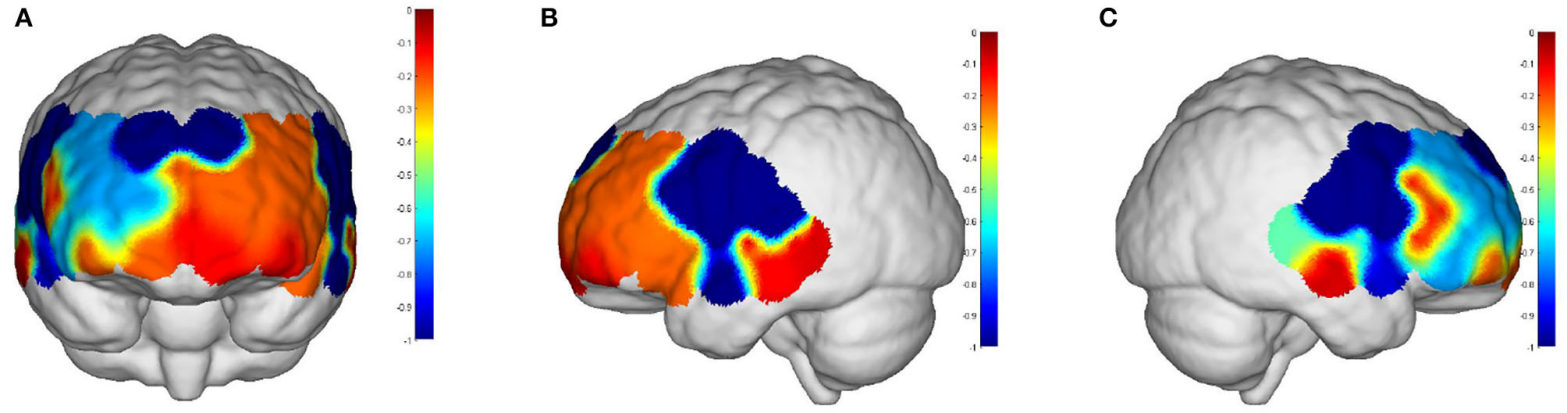
The *p-*values of task period in older adults compared to the young: **(A)** front view; **(B)** left view; **(C)** right view. The red areas are shown in prefrontal and DPLFC, which were *p* > 0.05.

Intervals III and IV were used to evaluate myogenic and neurogenic activation. Therefore, the following data were derived from the two intervals to examine the brain's activation level. The task state revealed higher WA in numerous brain regions than the resting state in both the elderly and the young people, indicating increased activation. FA and TA on the left and Broca on the right show statistically significant activation in left and right DLPFC elder cortical contrasts (*p* < 0.05) (Figure 5). In contrast, whereas WA was higher in both task and resting stages in young adults, this difference was not statistically significant (*p* > 0.05) (Figure 6). Comparing the task period activation between the two groups revealed no statistical significance, despite the fact that both older people had higher activation values than their younger counterparts (*p* > 0.05) (Figure 7).

#### Functional Connectivity Characteristics and Differences

**Figure 8:**
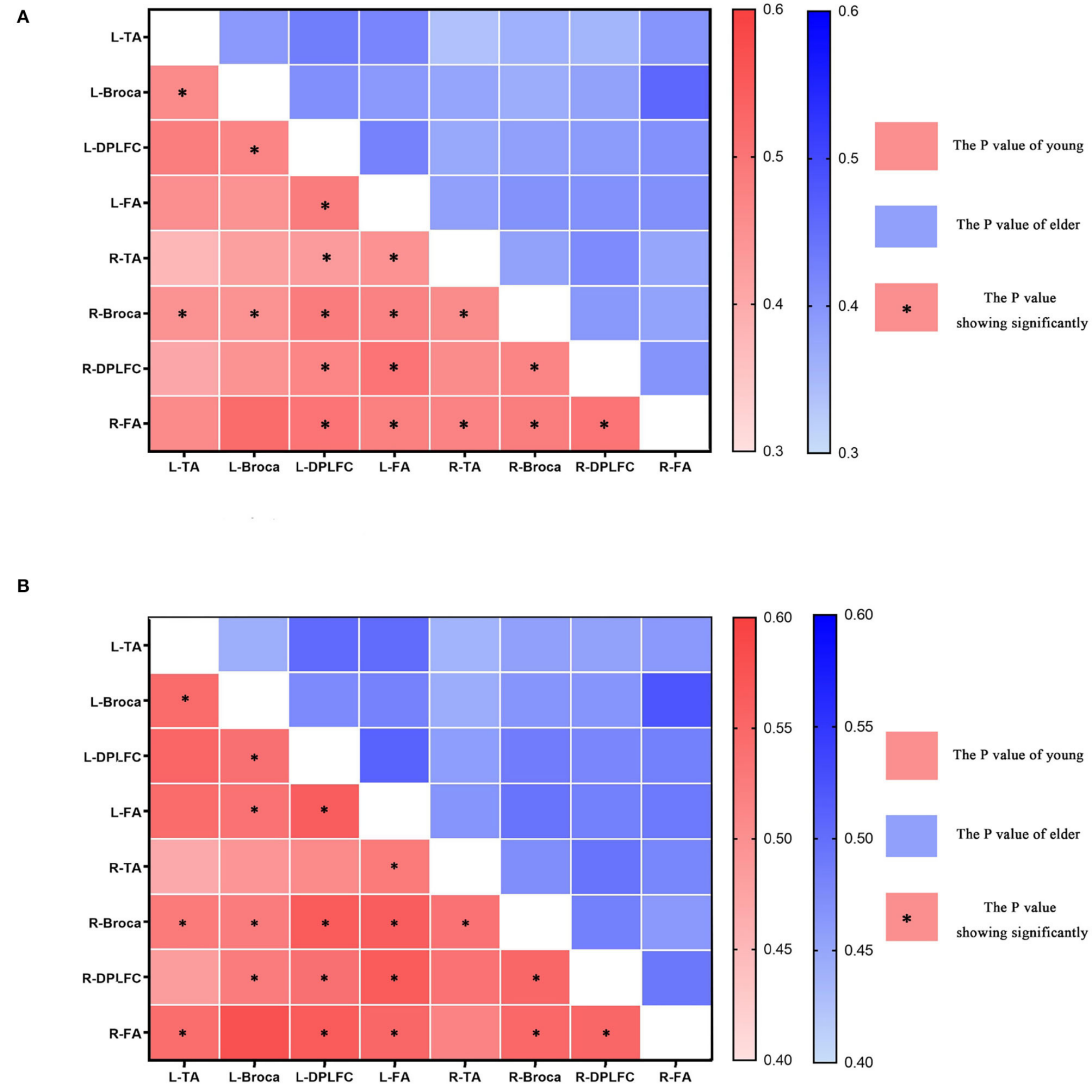
The *p-*values of different brain areas in young and elder people, L-x = L corresponding brain areas, R-x = R corresponding brain areas, * in red box indicates significant enhancement in the connectivity of young. **(A)** The connectivity in interval III. **(B)** The connectivity in interval IV.

In the functional connectivity of task states, comparison analyses were conducted for intervals III and IV (see Figure 8 and Table 2). Younger individuals demonstrated greater connections between different brain areas in both the left and right hemispheres during interval III (*p* < 0.05). The majority of these brain areas exhibited a statistically significant increase in connection (*p* < 0.05). In particular, the right Broca's area in young individuals was more connected to all other brain regions than in older individuals. In addition, the DLPFC and FA on the left demonstrated superior connectivity relative to the majority of the other brain regions. Young adults in interval IV demonstrated increased activation in multiple brain regions and similar activation in the right Broca's area (*p* < 0.05). Analysis revealed that statistically meaningful links were more prevalent in interval IV than in interval III. In contrast to interval III, left and right DLPFC connections were greater in the majority of brain regions. This result indicated that younger participants had higher functional connectivity between brain regions than older participants. This functional connection was established by synchronizing the fluctuations of various brain areas. Therefore, such functional connectivity meant that young people were better able to collaborate across diverse brain regions in order to collaboratively process task-related information and eliminate interference.

**Table 2:** The mean connection strength between different brain areas in intervals III and IV of young and elder people.

**The connectivity between different areas**	**Interval III**			**Interval IV**		
	**Y-mean**	**E-mean**	***P* value**	**Y-mean**	**E-mean**	***P* value**
Left TA-Left Broca	0.460	0.393	0.033^*^	0.546	0.443	0.010^*^
Left TA-Left DLPFC	0.482	0.429	0.077	0.554	0.504	0.102
Left TA-Left FA	0.451	0.419	0.257	0.544	0.502	0.127
Left TA-Right TA	0.376	0.337	0.125	0.468	0.438	0.232
Left TA-Right Broca	0.444	0.360	0.050^*^	0.527	0.456	0.021^*^
Left TA-Right DLPFC	0.406	0.354	0.074	0.483	0.454	0.239
Left TA-Right FA	0.461	0.398	0.102	0.541	0.462	0.022^*^
Left Broca-Left DLPFC	0.470	0.407	0.036^*^	0.540	0.475	0.007^*^
Left Broca-Left FA	0.444	0.393	0.110	0.536	0.482	0.041^*^
Left Broca-Right Broca	0.443	0.365	0.022^*^	0.525	0.465	0.018^*^
Left Broca-Right DLPFC	0.443	0.381	0.072	0.525	0.465	0.022^*^
Left Broca-Right FA	0.516	0.460	0.142	0.579	0.522	0.073
Left DLPFC-Left FA	0.487	0.423	0.026^*^	0.563	0.512	0.030^*^
Left DLPFC-Right DLPFC	0.469	0.389	0.005^*^	0.539	0.478	0.005^*^
Left DLPFC-Right FA	0.499	0.403	0.007^*^	0.564	0.483	0.004^*^
Left FA-Right FA	0.476	0.404	0.033^*^	0.551	0.490	0.044^*^
Right TA-Left Broca	0.421	0.376	0.179	0.492	0.443	0.070
Right TA-Left DLPFC	0.428	0.371	0.032^*^	0.507	0.457	0.027^*^
Right TA-Left FA	0.446	0.381	0.015^*^	0.526	0.465	0.050^*^
Right TA-Right Broca	0.456	0.380	0.004^*^	0.535	0.472	0.004^*^
Right TA-Right DLPFC	0.456	0.411	0.127	0.535	0.495	0.072
Right TA-Right FA	0.474	0.375	0.004^*^	0.515	0.478	0.180
Right Broca-Left DLPFC	0.487	0.384	0.000^*^	0.564	0.488	0.007^*^
Right Broca-Left FA	0.475	0.401	0.021^*^	0.563	0.495	0.018^*^
Right Broca-Right DLPFC	0.471	0.393	0.011^*^	0.551	0.483	0.018^*^
Right Broca-Right FA	0.483	0.380	0.004^*^	0.550	0.462	0.009^*^
Right DLPFC-Left FA	0.499	0.403	0.007^*^	0.564	0.483	0.004^*^
Right DLPFC-Right FA	0.495	0.399	0.003^*^	0.550	0.492	0.023^*^

*The p-values mean the comparison between the connection between elder and young people. Y and E indicate young and elder people;*

* *p < 0.05*.

## Discussion

Our experimental results were supported by the fNIRS technique, which showed differential patterns of cortical activations and functional connectivity between young and elder people during the performance of the Stroop task. In particular, the elderly demonstrated substantially greater activation of brain regions, including the DLPFC than the young. In contrast, in terms of functional connection, younger individuals had, on average, greater functional connectivity than older individuals. It was hypothesized that, to perform a task, the elderly required a greater activation of brain regions than the young. Young people's brains were less activated, but they were able to complete the task more effectively by activating more brain regions. In other words, older individuals were unable to coordinate the use of multiple brain regions to execute the job. They could only accomplish it by raising the arousal.

### Findings on the Stroop Test and the Result of fNIRS Between Different Ages

The analysis revealed that the DLPFC was one of the brain regions with the highest activation during task completion (Markowska et al., 2015; Parris et al., 2021). The Stroop task was a conflict inhibition in executive functions-focused test. In the shortest possible time, participants were asked to choose the correct response between two conflicting alternatives. Incongruent responses are slower than congruent responses, which demonstrate a conflict between the automatic reading tendency and naming colors under conscious control (Chen et al., 2013). Diverse parts of the brain were able to adapt to a wide range of challenges. There was evidence that the left DLPFC was more responsive to Stroop tasks. The Stroop task's pattern of selective attention was perfectly realized by the left DLPFC's implementation of top-down attentional regulation (Vanderhasselt et al., 2006). Consequently, in the context of sensitivity of both bilateral DLPFCs to the Stroop task, it was likely that the left side would be more responsive, consistent with our experimental findings. Not only did the WA of the left DLPFC increase during the actual job, but the WA of the right DLPFC also increased. It revealed that the bilateral DLPFCs of the elderly were simultaneously processing information and completing the executive function task while doing the Stroop task. Therefore, we reasoned that the activation of the DPLFC in elderly individuals occurred bilaterally at the time of activation.

Activation in the temporal area is believed to be connected with reading and language function activation (Luo, 1999; Ning, 2021). During the administration of the Stroop test, semantic competition represented both suppression of answers and elimination of interference. Interference occurs when visual information in a word conflict with color (e.g., a blue word and a yellow color) during a task requiring meaning evaluation. In contrast, interference was reduced when the meaning of the word and the color matched, although mental inertia from the prior test task interfered with judgements. Multiple areas of the temporal lobe were simultaneously engaged by the presence of both types of interference, resulting in semantic competition (Sturz et al., 2013; Britt et al., 2016; Piai and Knight, 2018; Gauvin et al., 2021). This investigation confirmed the hypothesis that the activation function of the brain varies with age. During the Stroop consistency/inconsistency assessment test, however, older participants processed information more slowly than younger ones, influencing the brain network that analyzes and categorizes cognitive processes. According to one article, the decreased ability of older individuals to suppress irrelevant information explains why older adults do worse on the Stroop task than younger participants (Stoltzfus et al., 1993). This also explains the fNIRS outcomes we observed. With this experiment, we gathered activation data for each brain region by measuring blood oxygen levels using NIR techniques. This allowed us to neurophysiologically identify the regions of the temporal lobe from where this semantic conflict originates.

In a previous study involving young and elder participants, greater activation in bilateral DPLFC and anterior ventral lateral prefrontal cortex during the Stroop test was the primary finding. In accordance with the fNIRS findings, the bilateral DLPFC, FA, TA on the left, and Broca regions revealed higher activation in the elderly patients. Nonetheless, we noticed a clear trend from the baseline (before to the start of the intervention) to the post-task, in which there was no significant difference between the comparison of young and older participants during the task time.

### Increasing Age was Accompanied by More Neurological Problems

Changes in functional connectivity patterns identified in younger adults using fNIRS revealed that it not only included the DPLFC but also other regions of the brain, such as the right Broca and left FA, which was much stronger in the young compared to the elderly people. It indicated the increased interhemispheric functional integration was strongly associated with the increased workload of brain, which was consistent with previous studies (Laguë-Beauvais et al., 2013). From a neurobiological point of view, it has been described that activities with different intensities trigger the release of norepinephrine, dopamine, and acetylcholine from the nucleus, which may in turn be caused by an increase in the general physiological activity of the organism (Burin and Kawashima, 2021). These nuclei are functionally connected to the hippocampus and prefrontal cortex. It is closely associated with executive functions, especially those involved in Stroop tasks and related to physical activity. As activity decreases in older adults due to reduced physical function, the structural and functional connectivity decreases simultaneously. In addition, older adults appear to be unable to suppress extraneous information that may interfere with the completion of specific goals. As a result of atrophy and degradation of the brain's white and gray matter, the functional connectivity of the elderly decreased significantly with age (Zhang et al., 2013). It has been experimentally proven that a decrease in gray matter volume with aging reduced the activity of brain regions directly associated with executive functions (Takeuchi et al., 2012). Simultaneously, the function of white matter in cognitive performance in older adults was gradually acknowledged (Wolf et al., 2014; Takeuchi et al., 2017; Li et al., 2018). The decrease in brain capacity produced by atrophy of the white matter resulted in slower processing of information and slower motor speed, which may have an effect on activity performance.

Considering the limitations of this study, in addition to cognitive abilities, the Stroop test also demands physical functions to execute the button pushing task. Consequently, this would necessitate activation of additional brain regions beyond those described in this work, which includes the spinal cord, brainstem, cerebellum, basal ganglia, and motor cortex, among others. Besides, our experiment did not add neutral stimulus in the Stroop task, which was one of the limitations that should be prevented. In addition, the DLPFC serves as a processing hub for a variety of perceptual functions, where it was possibly due to a contingency or a repetition effect. Although fNIRS was utilized in this study to determine which brain region the Stroop task activates, this activation may be related to other factors shown in additional trials. For instance, the surroundings should be as calm as possible, with as few interfering objects as feasible. In addition, subjects must be attentive and free of distractions during the experiment. Nonetheless, it is important to conduct appropriate fNIRS exams on larger populations and in a greater number of disorders.

## Conclusion

This study provides strong evidence that greater levels of DLPFC and Broca boost functional connectivity in the brain compared to Stroop tests in young and elderly persons. Although the elderly had greater cortical activation than the young people, the young had stronger connectivity in the majority of brain regions. Similarly, with growing age, the rate and accuracy with which elderly adults complete activities declines. Elders are unable to properly utilize the frontotemporal lobes to execute essential cognitive tasks, according to the hypothesis.

## Data Availability Statement

The raw data supporting the conclusions of this article will be made available by the authors, without undue reservation.

## Ethics Statement

The studies involving human participants were reviewed and approved by the Ethics Committee of the Third Hospital of Sun Yat-sen University hospital. The patients/participants provided their written informed consent to participate in this study. Written informed consent was obtained from the individual(s) for the publication of any potentially identifiable images or data included in this article.

## Author Contributions

WH, XL, HX, TQ, YZ, LS, Z-MT, and ZD worked together to complete the manuscript. ZD, YZ, Z-MT, and LS assisted WH and XL in document retrieval and screening. HX and TQ provided statistical assistance and provided opinions on grammar and rhetoric. All authors contributed to the article and approved the submitted version.

## Funding

This work was supported by the National Key Research and Development Program of China (Grant No. 2020YFC2004205), the Key Realm R&D Program of Guangzhou, China (Grant No. 20200703007), and Shenzhen Science Technology Project (Grant No. JSGG20201102145602006).

## Conflict of Interest

The authors declare that the research was conducted in the absence of any commercial or financial relationships that could be construed as a potential conflict of interest.

## Publisher's Note

All claims expressed in this article are solely those of the authors and do not necessarily represent those of their affiliated organizations, or those of the publisher, the editors and the reviewers. Any product that may be evaluated in this article, or claim that may be made by its manufacturer, is not guaranteed or endorsed by the publisher.
